# Inferring detailed space use from movement paths: A unifying, residence time‐based framework

**DOI:** 10.1002/ece3.3321

**Published:** 2017-09-12

**Authors:** Dror Kapota, Amit Dolev, David Saltz

**Affiliations:** ^1^ Mitrani Department of Desert Ecology Jacob Blaustein Institutes for Desert Research Ben‐Gurion University of the Negev Midreshet Ben‐Gurion Israel; ^2^ Science Division Nature and Parks Authority Jerusalem Israel

**Keywords:** animal movement, movement path analysis, first passage time, foraging behavior.

## Abstract

The residence time is the amount of time spent within a predefined circle surrounding each point along the movement path of an animal, reflecting its response to resource availability/quality. Two main residence time‐based methods exist in the literature: (1) The variance of residence times along the path plotted against the radius of the circle was suggested to indicate the scale at which the animal perceives its resources; and (2) segments of the path with homogeneous residence times were suggested to indicate distinct behavioral modes, at a certain scale. Here, we modify and integrate these two methods to one framework with two steps of analysis: (1) identifying several distinct, nested scales of area‐restricted search (ARS), providing an indication of how animals view complex resource landscapes, and also the resolutions at which the analysis should proceed; and (2) identifying places which the animal revisits multiple times and performs ARS; for these, we extract two scale‐dependent statistical measures—the mean visit duration and the number of revisits in each place. The association between these measures is suggested as a signature of how animals utilize different habitats or resource types. The framework is validated through computer simulations combining different movement strategies and resource maps. We suggest that the framework provides information that is especially relevant when interpreting movement data in light of optimal behavior models, and which would have remained uncovered by either coarser or finer analyses.

## 1 INTRODUCTION

Animals move to accommodate changes in the availability of their resources over time and space (Sims, Witt, Richardson, Southall, & Metcalfe, 2006; Weimerskirch, Pinaud, Pawlowski, & Bost, 2007). Studying this process requires the estimation of statistical measures from empirical movement paths, for identifying patterns or fitting mechanistic movement models (Buderman, Hooten, Ivan, & Shenk, 2015; Dalziel, Morales, & Fryxell, 2008; Morales, Haydon, Frair, Holsiner, & Fryxell, 2004; Patterson, Thomas, Wilcox, Ovaskainen, & Matthiopoulos, 2008). These patterns may be complex, depending on the cognitive abilities of the animal and the structure of the habitat. One such a statistical measure is the residence time (Barraquand & Benhamou, 2008; Knell & Codling, 2012; Sur et al., 2014), which is time spent within confined areas along the path. Other common measures are the mean‐squared displacement (Johnson, Milne, & Wiens, 1992), overall tortuosity of the path (Benhamou, 2004), distributions of step lengths, turning angles, and movement speeds (Turchin, 1998). While the mean‐squared displacement provides holistic characterization of the movement (directional or bounded movement, autocorrelated movement etc.), the other measures describe more detailed behaviors such as changes in the shape or speed of movement in response to the availability of resources (Fryxell et al., 2008).

The residence time captures changes in both the shape and the speed of movement. For example, regardless of whether the animal moves more tortuously in areas with high food availability or simply slows down, it would spend more time in a predefined unit of area. Moreover, the allocation of time for different activities is an important element in optimal behavior theory, so using time as the main variable may also facilitate the interpretation of movement in this context.

The key task in movement analysis is using the statistical measures in order to characterize distinct behavioral modes in the path (heading toward a patch, searching food within the patch, resting etc.) and assign these modes and their switching probabilities to various environmental elements (Dalziel et al., 2008; Getz & Saltz, 2008; Jonsen, Flemming, & Myers, 2005; Morales et al., 2004; Pinto & Spezia, 2015; Sur et al., 2014). However, such analyses can be performed at an infinite range of resolutions, and as behavioral modes are performed at a variety of spatiotemporal scales (Fauchald & Tveraa, 2006; Fritz, Said, & Weimerskirch, 2003; Fryxell et al., 2008), the ability to discern these modes depends strongly on the resolutions chosen. There is a need to identify the level of resolution that will capture the main motives of the animal's behavior.

Fauchald and Tveraa (2003) suggested a residence time‐based method for identifying the scale of area‐restricted searches, and Barraquand and Benhamou (2008) suggested using the residence time to characterize distinct behavioral modes in the path. These methods can help identifying responses to resource availability under the assumption that animals spend more time within profitable places than elsewhere, but require some modifications and a proper integration. Here, we integrate these methods into a single analysis framework with two steps: (1) determining distinct resolutions that reflect the main scales (as there may be more than one) at which an animal performs intensive search; and (2) at these resolutions, identifying confined places to which the animal paid multiple visits and performed intensive search, and for each place estimating the mean duration spent per visit and the number of revisits. The first step indicates how animals perceive and respond to a multiscale resource environment, and provides the appropriate resolution at which the analysis should proceed. The second step zooms in at a specific scale, indicating how animals use patches of different resource types at the given scale. We validate the performance of analysis using computer simulations.

## 2 REVIEW OF PREVIOUS METHODS AND INTRODUCING THE FRAMEWORK

The first‐passage time (FPT) is a measure in statistical physics, defined as the time it takes for a moving particle to reach a certain location for the first time (Montroll, 1969). By delineating a virtual circle of a radius *r* around each location along a recorded movement path of an animal, the FPT is defined as the time it took the animal to reach the perimeter of the circle for the first time. The residence time (RT) is then defined as FPT_backward_ + FPT_forward_, namely the total time the animal spent within the virtual circle from first entry to first exit (Barraquand & Benhamou, 2008; Fauchald & Tveraa, 2003; Knell & Codling, 2012). In this approach, locations with high RT represent locals in which the animal performed an area‐restricted search (ARS), that is, an intensive search within a confined area, characterized by a tortuous or slow movement.

When the size of the virtual circle matches the size of the area covered by ARS, the difference in RT between circles containing ARS to all other circles will be the largest, and therefore, variation in RT will be maximized. On this basis, the variance of log(RT) can be plotted for circles of different *r* values to obtain a variance‐scale curve [the log transformation makes the variance independent of the mean—Fauchald and Tveraa (2003)]. A peak in the variance‐scale curve appears for the radius that matches the ARS. The radius associated with this peak was suggested to quantitatively indicate the characteristic size of a food patch. This suggestion was later criticized by Barraquand and Benhamou (2008), stating that for varying but close patch sizes, or for certain forms of intensive search, the radius associated with a peak in the variance‐scale curve is unreliable as a quantitative estimator of patch size.

Here, we introduce several improvements to the variance‐scale curve method. Fauchald and Tveraa (2003) examined the variance‐scale curve only for the case of one distinct ARS size and therefore a single peak. They indeed discussed the possibility of several distinct ARS scales, occurring when several distinct patch sizes exist, or when patches are organized in a hierarchical manner (Fauchald & Tveraa, 2006). But, they treated this issue by performing a nested analysis—calculating the variance‐scale curve for the whole path, using the radius associated with a peak to calculate RTs, choosing path segments with high RTs, and then calculating new variance‐scale curves for these segments. Nevertheless, when several distinct ARS scales exist, the variance‐scale curve itself should show several distinct peaks, reflecting a multiscale response for the entire path. We validate this multipeak existence by studying variance‐scale curves of simulated hierarchical ARS movements, and compare the performance of the method using variance of log(RT) against using the coefficient of variation in RT.

Another issue raised by Fauchald and Tveraa (2003) is that the pattern formed in the variance‐scale curve is shaped by both the distribution of resources and the response of the animal to this distribution. Using simulations, we separate these two effects, such that patterns in the variance‐scale curve can be assigned directly to the behavior. We also address the critique of Barraquand and Benhamou (2008) on the method by evaluating the performance of the curve in estimating ARS scales for simulated paths where ARS is variable yet found within distinct scale domains.

Following the variance‐scale method, Barraquand and Benhamou (2008) suggested a method to separate the path into segments constituting homogeneous movement bouts, in terms of their RT values. These homogeneous movement bouts can be interpreted as different behavioral modes, such as ARS, exploration, or directed fast movement. The RT in this case may contain not only FPT_backward_ + FPT_forward_, but also additional forward and backward path segments found within the circle, as long as the time outside the circle between these segments is shorter than some predefined threshold. These homogeneous movement bouts can then be correlated with environmental data providing insight regarding the response of the animal to its environment and resources.

Barraquand and Benhamou (2008) did not suggest any robust way of choosing a proper resolution for their analysis. They suggested performing the analysis at several resolutions, and choosing what seems to be most reliable. This approach does not account for the possibility of hierarchical ARS at several distinct scales. Another important issue is that the series of path segments has no explicit spatial interpretation. Two segments constituting ARS may have been recorded within the same place where the animal revisited twice, or at two distinct places. Moreover, as Barraquand and Benhamou (2008) suggested adding forward and backward path segments found within the circle into the RT, two distinct segments recorded within the same place may be, in part, pseudoreplication. The two latter issues were later treated by giving the series of residence times an explicit spatiotemporal representation within the framework of kernel‐based utilization distributions (Benhamou & Riotte‐Lambert, 2012); however, no appropriate solution was provided for choosing the appropriate resolution. Identifying places in which the animal revisited and performed ARS several times is important for mapping areas of interest and correlating them with environmental variables (Bar‐David et al., 2009; Benhamou & Riotte‐Lambert, 2012; Riotte‐Lambert, Benhamou, & Chamaillé‐Jammes, 2013). In terms of habitat preference and use, several visits to several distinct places having the same environmental properties may have a different meaning than several revisits to the same place. We suggest an analysis with two steps: First, the variance‐scale curve is used for identifying the meaningful scale domains in the path; second, at these scale domains, we describe a residence time‐based algorithm that identifies spatially distinct ARS places and calculates the mean visit duration, and the number of revisits in these places. These variables should, later on, be confronted with environmental data. We validate the performance of the analysis using computer simulations.

## 3 METHODS

### 3.1 Identification of ARS scales

**Figure 1:**
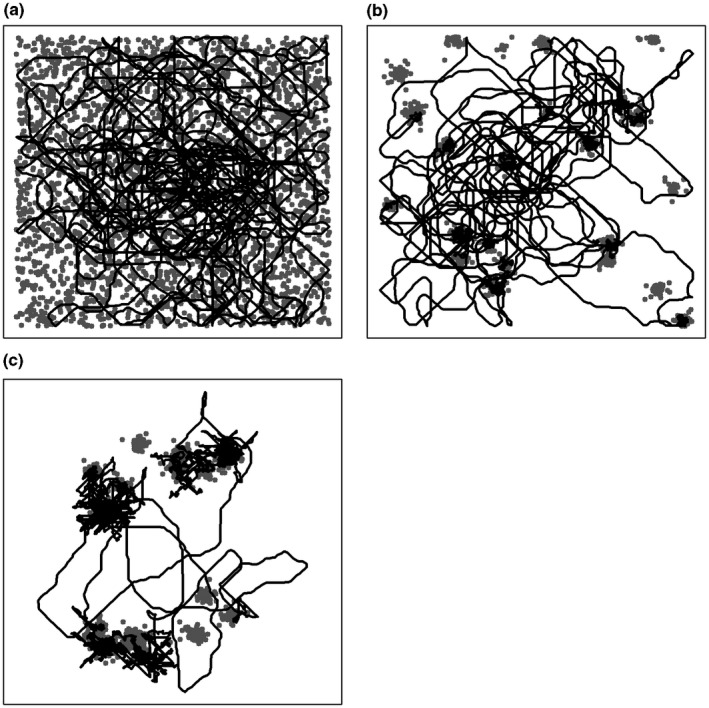
Examples of simulated resource maps (gray dots) and movement paths (black lines). Scattered resource map and simple search (a); patchy resource map and area‐restricted search (ARS) (b); hierarchical resource map and hierarchical ARS (c)

To study the shape of the variance‐scale curve for ARS movements at one to several scales, and to separate the effects of resource distribution and the movement behavior on this shape, curves were calculated for five different simulated combinations of resource maps and movement strategies (see details in the simulation model section below and Appendix S1 Matlab code is found as online supplementary information ‐ Appendix S3): (1) simple search for scattered resources; (2) simple search for patchy resources; (3) ARS for patchy resources; (4) ARS for hierarchical patchy resources; and (5) hierarchical ARS for hierarchical patchy resources. Simulated paths were resampled (one of each ten locations) to mimic the frequency at which real paths are sampled by a GPS device. Variance‐scale curves were calculated by calculating for each radius the residence time of each location along the path [residence time calculation follows Barraquand and Benhamou (2008)], and then calculating variation among locations for each radius. Each variance‐scale curve was calculated twice, for comparison: as the variance of log(RT) against *r* and as the coefficient of variation in RT against *r*. For each curve, we measured the number of peaks, the strength of the peak signature, and the radii of ARS. The latter was compared with direct estimations measured from the visualized paths. This was possible as the simulations, albeit stochastic in nature, created ARS within one or two distinct scale domains, which could be distinguished visually (Figure 1). For each simulated scenario, simulations were run 30 times and variance‐scale curves were calculated. In two scenarios (ARS for patchy resources and ARS for hierarchical patchy resources), the pattern was not entirely consistent—additional shallow peaks appeared in several runs. We therefore simulated and analyzed additional 30 runs for each of the two scenarios, and verified using random permutations that the rate of obtaining an additional peak does not vary with sample size.

### 3.2 Identifying revisited ARS places

Revisited ARS places are spatially confined and distinct areas in which the animal performed ARS more than once. They are supposed to be places of interest for the animal, such as food patches. The location and size of ARS places are unknown, but can be inferred indirectly from the recorded (known) locations of the animal.

The algorithm has four steps illustrated in Fig. S3: (Matlab code is found as online supplementary information ‐ Appendix S3) (1) calculating RTs along the path; (2) filtering non‐ARS path segments; (3) identifying spatially distinct ARS places; and (4) choosing a representative location for each ARS place identified. In step (1), the algorithm traces, for each recorded location, all backward and forward segments of the path passing within the boundaries of its circle (i.e., backward and forward RT segments), recording their durations separately. Each circle around each location thus has a revisit history—a series of recorded revisits within the circle, their timing, and duration (see Fig. S3 for visual illustration).

In step (2), revisited ARS places are distinguished from other places the animal crossed more than once, while *en route* and recorded path segments of the latter are filtered out. As a filtering criterion, it is possible to use the mean duration per visit or the total duration spent around recorded locations, as both should distinguish the long ARS revisits from the fast occasional ones; here, we examined and compared both. As a filtering threshold, we suggest the mean or median (whichever is largest—as these distributions are strongly left‐skewed) of the distribution of the criterion chosen.

In step (3), spatially distinct ARS places are identified by considering the remaining locations as the nodes of a graph, in which connections (edges) exist if two locations are found within the circles of each other. ARS places are defined as connected components of this graph; that is, subgroups in which any two nodes are indirectly connected to each other (see Fig. S3 for visual illustration and additional explanations). These connected components are identified using a recursive depth‐first search algorithm (Cormen, Leiserson, Rivest, & Stein, 2001).

Step (4) estimates the exact location of each place, its mean visit duration, and number of revisits. The mean visit duration and number of revisits to recorded locations that are assigned to the same ARS place constitute various portions of the full revisit history of this place. Therefore, one of the locations in each ARS place should be chosen to represent the central location of the place and its revisit history. Choosing this location was done using the same two criteria used for filtering—we examined both the location with the longest mean duration per visit and the location with the longest total duration spent within its circle (Fig. S3).

We used the simulation of simple ARS for patchy resources to evaluate the performance of the algorithm including the examination of filtering and choosing criteria. As the simulated resource maps and movement modes are simple and well defined, we could identify ARS places visually from each realized resource map and path. We could therefore compare the ARS places identified by the algorithm to those identified visually. This allowed us to spot failures and erroneous identifications of the algorithm: places that were erroneously united because of their proximity, stopping places, which are not ARS places (these have long visit duration and were therefore filtered in when the mean visit duration was used for filtering and choosing locations), and places with occasional, multiple, and short revisits (these have long total time and were therefore filtered in when the total time was used for filtering and choosing locations). The simulation was run 30 times to ensure that the algorithm identifies most of the ARS places successfully, and to properly estimate error rates.

### 3.3 The simulation model

Simulations were run for 60,000 time steps on resource maps of 1,000 × 1,000 cells. Three spatial structures of food items were created as follows: scattered, simple patchiness, and two‐level hierarchical patchiness (Figure 1). Food items (for scattered resource map) and patch centers (for patchy resource maps) were scattered over the matrix randomly. The number of food items in a patch (and the number of patches in a clump for hierarchical patchiness map) and their distances from patch (or clump) center were drawn from normal distributions, and their direction was drawn uniformly. Food items re‐emerged gradually after being exploited. More simulation details and parameter values are given in Appendix S1—simulation details.

The general movement model used is a correlated random walk biased toward or away from a predefined target location. Step length is one cell, and the direction at time *t*, θ_*t*_, is drawn from a normal distribution:

(1) $$\theta_{t}∼N\left(\theta_{t-1},v\right)$$

$$v=v_{b}\left[1\pm p*\cos(\theta_{t-1}-\omega_{t-1})\right]$$

where θ_*t*−1_ is the previous direction, *v*_*b*_ is the basic variance, *p* is the level of attraction to a target location, and ω_*t*−1_ is the current direction to the target location (Benhamou & Bovet, 1989). The attraction parameter *p* ranges from 0 (no attraction to the target location) to 1 (complete attraction). The sign before *p* is minus for attraction, or plus for repulsion. Different combinations of *v*_*b*_ and *p* yield a variety of movement behaviors: from directed movement toward the target to concentrated ARS around the target to widely dispersed exploration behavior (see Appendix S1—simulation details).

Three foraging strategies were simulated (Figure 1). In the first strategy, a simple search, the forager explores the map and consumes food items whenever they are within a predefined perceptual range. In the second strategy, a simple ARS, the forager explores the map equipped with a limited perceptual range, until it first encounters a food item. Then, it starts performing an ARS, which continues as long as additional food items are found, and terminates once a predefined giving‐up time elapses without finding any food item (Charnov, 1976; Krebs, Ryan, & Charnov, 1974). Then the forager leaves the patch and reverts to the exploration mode. The third strategy, the hierarchical ARS, is the same as the simple ARS until the forager leaves a patch. Once leaving a patch, the forager starts searching for another patch in the vicinity of the patch it just left by performing repeated forays radiating away from the patch and back toward it, until finding a new patch or until a predefined giving‐up time elapses (Conradt, Zollner, Roper, Frank, & Thomas, 2003).

## 4 RESULTS

### 4.1 Identification of ARS scales

**Figure 2:**
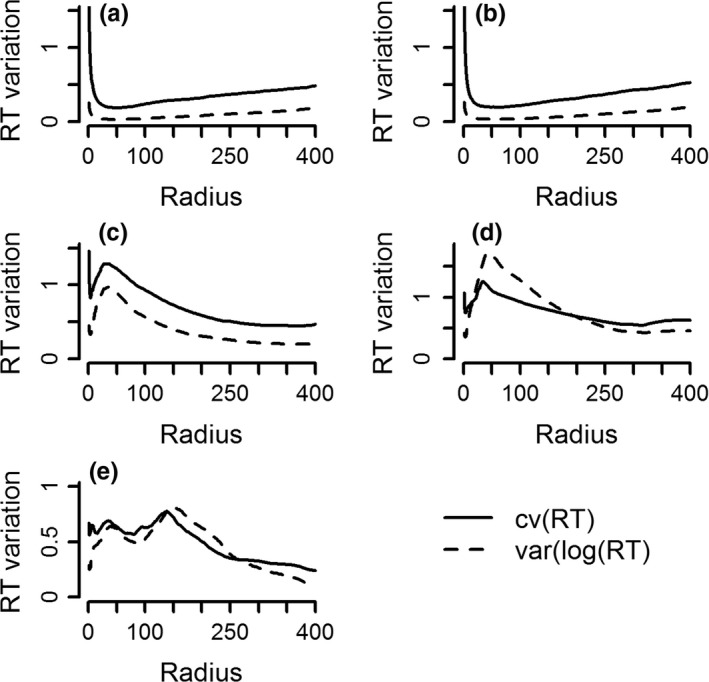
Variance‐scale curves of simulated paths. Simple search for scattered resources (a); simple search for patchy resources (b); area‐restricted search (ARS) for patchy resources (c); ARS for hierarchical resources (d); hierarchical search for hierarchical resources (e). The acronym “var” stands for variance and “cv” stands for coefficient of variation

The number of peaks in the variance‐scale curve indicates the number of distinct scales to which the forager responds. A simple search performed on any resource map (scattered and patchy) yielded low and constant variance‐scale curve, peaking only at *r* = 0 because of resting stops, in all 30 × 2 simulation runs (Figure 2a,b). No peaks were formed if the animal allocated time uniformly over its path, even if the resources were organized in a patchy manner. An ARS performed in a patchy environment yielded a variance‐scale curve with one distinct peak in 25 simulation runs, and two shallow peaks in five simulation runs (Figure 2c and Table S1). When ARS behavior was performed on a resource map with hierarchical patchiness, 23 simulation runs produced a single peak, and seven runs produced two rather shallow peaks. These additional shallow peaks emerged when patches were close, and the simulated animal moved between them by chance. Hierarchical ARS performed on hierarchical patchy resources produced 2–4 distinct peaks in 29 of 30 simulation runs (Figure 2e and Table S1). Using variance of log(RT) or the coefficient of variation in RT, produced similar results for medium to large radii, while for small radii, peaks that appear clearly with the coefficient of variation are overflattened or totally absent by the log transformation (Figure 2). The log transformation tends to produce slightly larger estimates for the radius of ARS than the coefficient of variation, and in most of the cases, both fall slightly above the range estimated visually (Table S1). The variance‐scale curve thus identifies qualitatively the scale domains at which ARS is performed, but as a quantitative estimator may be biased high. The bias is larger when the variance of log(RT) is used. In all cases, as the radius increases, the variance eventually decreases because larger circles contain more heterogeneous path segments, so RT values become more similar.

### 4.2 Identification of revisited ARS places

**Figure 3:**
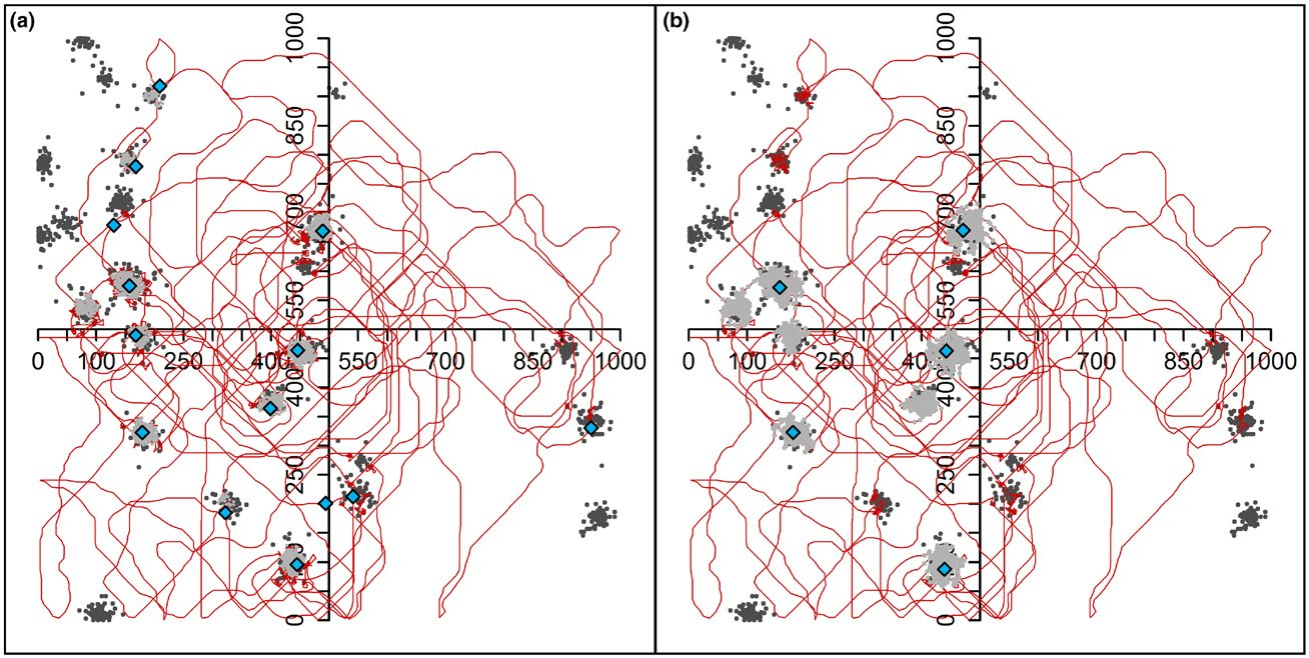
Example of output of the algorithm identifying area‐restricted search (ARS) places for simulated paths based on two criteria for filtering out locations and choosing representative locations (Simulation No. 7 in Table S1). The mean visit duration (a) and the total accumulated time (b). The resource map is colored deep gray; the simulated path is colored red; groups of locations identified to be within ARS places are colored light gray, and representative locations for those ARS places as displayed as blue diamonds. In the presented case, when using the mean visit duration as filtering criterion, the algorithm successfully identified most of the ARS places, but identifies also one stopping site where one long visit occurred (the diamond in [0, 200] in a) and erroneously uniting one site with another, adjacent site. When using the total accumulated time as filtering criterion, the algorithm overlooks nine ARS places, where small number of visits occurred, and erroneously uniting three sites with other adjacent sites

The algorithm performed slightly different when using the mean visit duration versus total accumulated time as a criterion for filtering out non‐ARS path segments and choosing the recorded location representing each ARS place. Using the mean visit duration as a filtering and choosing criterion, the algorithm identified most of the food patches in which ARS were performed in the simulated paths (Figure 3 and Table S2—compare with ARS places identified visually). However, the algorithm occasionally identified also places where a single long stop (not ARS) occurred, which can be avoided by ignoring places with only one visit. Using the total accumulated time as a filtering and choosing criterion, the algorithm overlooked places with one or two long ARS visits, as their total accumulated time is relatively low, and hence consistently underestimated the number of ARS places. On the other hand, the algorithm occasionally highlighted places that were revisited multiple times for short durations (not ARS). Under the two criteria, when ARS places were very close to each other, locations from one place encircled locations from the other place, leading the two places to be erroneously identified as one (see Figure 3). This error was slightly higher when using the total accumulated time, as many locations at the margin of the ARS places were revisited multiple times and not filtered out.

## 5 DISCUSSION

### 5.1 Identification of ARS scales

The variance‐scale curve indicates the scales at which animals respond to their environment. Considering resources as the only movement driver in our simulations, the curves discriminated between distinct scale domains formed in the path when the animal responded to resources organized in a spatially hierarchical manner (Fauchald & Tveraa, 2006). The pattern formed in the path, and hence the shape of the curve, is much more sensitive to the behavioral model than to the resource map itself: Simple movement strategies yielded simple curves when performed on a complex resource map in the majority of simulations runs. If a complex pattern in the path does not tend to emerge spontaneously, purely on basis of a complex resource map, the variance‐scale curve is indicative of the behavior itself and can discriminate complex behaviors from simple ones.

The way one presents variation in RT is important. The use of log transformation (Fauchald & Tveraa, 2003) relies on the assumption that RT values are distributed log‐normally (Limpert, Stahel, & Abbt, 2001), and its tendency to overflatten the peak pattern at small radii may indicate the deviation of RT distribution from log‐normal at small radii. The use of coefficient of variation may be preferred in cases where small scales are particularly important. The peak pattern cannot be regarded as a precise quantitative estimator of ARS or patch size; rather it provides indication for distinct scale/s at which the animals perceives and reacts to resources. Patch size in natural environments is characteristically variable within each scale domain, making precise estimations impossible. On the other hand, such precision is not necessary for understanding the spatial scales at which animals view their environment.

### 5.2 Identifying revisited ARS places

The mean visit duration and number of revisits provide a deeper view into the animal's behavior and relationships with the environment, compared with more basic statistical measures such as the residence time itself, tortuosity, or turning angles. The mean visit duration within confined ARS places indicates the time spent within a food patch per visit—an important variable in optimal foraging theory characterizing patch use strategies under temporal depletion and uncertainty conditions (Brown, 1992; Charnov, 1976; Mcnamara, 1982). The revisit frequency serves as a complementary measure indicating the level of attraction to certain places given the mean and variance of their profitability, and is useful especially when temporal patch depletion limits the duration per visit (Benhamou & Riotte‐Lambert, 2012). Revisit frequency also indicates spatial knowledge, memory, and the patches’ renewal rate. These measures can hence be used to investigate movement data in light of optimal behavior models. Moreover, the association between the two measures is a signature of how animals use a certain habitat or resource type. For example, places with high food density may be featured by many long revisits, while places in which fast and high reward is obtained (such as water sources) may be featured by many short revisits. Places which are not replenished or vary greatly in their profitability may be featured by few long revisits.

In addition to summarizing the revisit history of ARS places, it may be of interest to examine it over time: periods when a place is visited more intensively than other periods or change in visit durations over time hint on the dynamics of resources. The distribution of between‐visit periods hints on the knowledge the animal has regarding profitable places. Knowledge should result in a trap‐lining behavior, where certain places are systematically revisited, but are avoided for some period after each visit to accommodate temporary food depletion. We give a simple example for such analysis applied on simulated knowledge‐based movement in Appendix S2.

Three technical points were highlighted by the results and should be addressed for any dataset before the analysis is performed: (1) The differences between the two criteria for filtering out locations and choosing locations to represent ARS places are important. If the goal is to identify ARS places, the mean visit duration should be the favored criterion. But in some cases, places that were relatively briefly revisited multiple times are meaningful, so the goals of the specific study should direct the choice of a criterion. Alternatively, the analysis may be performed twice using both criteria, and the results can be compared; (2) adjacent, distinct ARS places may be erroneously identified as one, especially when patches are very close to each other. To overcome this problem, after applying the analysis, the connected components should be visualized, and components which appear to have a radius twice larger than the characteristic radius of ARS places (as indicated by the variance‐scale curve) should be split manually. The problem becomes crucial for radii, which are large relative to the whole occurrence area of the animal, making the method inappropriate for such cases, for example, for studying home‐range behavior. For such purposes, utilization distributions are more appropriate; (3) performing the analysis using characteristic radii smaller than the actual radius of ARS bears a serious problem of erroneously counting several in–out movements as several revisits. This problem may be overcome by summing into the RT additional time segments within the circle, up to some threshold of time outside (Barraquand & Benhamou, 2008). However, choosing this threshold demands a priori knowledge of the relevant temporal scales. Optionally, given the relevant spatial scale, a radius larger than the actual mean radius of ARS places (as indicated by the variance‐scale curve) can be used. When several paths are analyzed and their variance‐scale curves indicate a range of radii, we suggest using the upper limit of this range for further analysis.

## 6 CONCLUSIONS

While the variance‐scale curve gives a holistic view of the spatial behavior, the visit durations and number of revisits are scale‐ and habitat‐specific, and provide information regarding the detailed space use. This information is remains hidden if only resource selection functions (Manly, 2002) or other coarse measurements of space use are used. Increasing the resolution may reveal new information on the way animals actually use their environment. Alternatively, using a highly detailed statistical description of the movement would conceal this information within the details. Using a mesoscale resolution, our RT‐based framework enables to extract from relocation data the specific information needed for correlating spatial behavior with the structure of the environment.

Nevertheless, our framework assumes that animals respond to resource abundance and distribution in a way that maximizes time within profitable places. Different associations between resources and movement could manifest themselves differently in terms of the RT. For example, an animal having a good knowledge of the location of food items could perform short and straight in–out movements into a food patch, such that peaks in the variance‐scale curve would not indicate ARS size or patch size. Choosing the proper scale using the variance‐scale method in such a case is problematic, but the revisit analysis would still be indicative on its own. Such associations between movement and resources should emerge when resources interact with other factors causing movement—reproduction prospects, conspecific attraction, and predator avoidance (Creel & Winnie, 2005; Creel, Winnie, Maxwell, Hamlin, & Creel, 2005; Valeix et al., 2009). The analysis presented here was focused on interactions with relatively stationary objects, unlike conspecifics, predators, or moving prey items. However predation risk and competition can be viewed as an integral part of patch quality, and applying the framework on such scenarios is still possible. Prior to its use, one should consider which motivations for movement the animal has other than food. Revisiting patterns should be judged differently, but will help inferring how different factors govern the way resources are visited and used.

## DATA ACCESSIBILITY

Matlab analysis code is uploaded as the online supporting information. Sample data will be archived on Dryad upon acceptance.

## CONFLICT OF INTEREST

The authors declare no conflict of interest.

## AUTHOR CONTRIBUTIONS

Dror Kapota, Amit Dolev, and David Saltz developed and integrated the methodological approach; Dror Kapota and David Saltz wrote the manuscript.
