# Supplementary material for: Inferring detailed space use from movement paths: A unifying, residence time‐based framework
Source: Ecol Evol. 2017 Sep 12;7(20):8507–14. doi: 10.1002/ece3.3321 (PMC5648670; doi:10.1002/ece3.3321)
Supplement: Supplementary file 1 [file ECE3-7-8507-s001.docx]

**Figure S3:** Illustration of the algorithm for identifying revisited ARS places. In (A) an illustrated path displaying two ARS places was sketched. Locations were recorded each arbitrary time unit. For each recorded location in the path, all backward and forward segments of the path passing within the boundaries of its circle were recorded. For illustration in (A), the sixth location was encircled, and two path segments were colored red: the segment that includes the location itself, and one additional, forward segment passing within the boundaries of the circle. In (B), concentrating on the lower half of the path, all locations that were visited twice or more are colored red, with their visit durations written aside. Next, non-ARS path segments are filtered out. In (C) all locations with mean visit duration of less than 4 were filtered out (re-colored blue). For setting the threshold value for real cases see main text. In (D) the entire path is shown again, with all locations having two or more visits and mean duration longer than 4 colored red. These “red locations” should be divided to spatially distinct ARS places. In (E) locations were sketched with their circles. Two locations are connected directly when encircling each other, and indirectly connected when there is a path of directed connections between them. Groups of locations defined as connected components – where any locations are indirectly connected, were assumed to represent distinct ARS places (see main text for the problem of distinct but very close ARS places, which are mistakenly united). Lastly, one of the locations in each group should represent the entire ARS place by means of its central location, number of revisits and mean visit duration. In (F) the location with the longest mean visit duration was chosen, colored purple, and its circle was sketched. This location is found roughly in the middle of the group, so its circle captures the majority of the three convoluted path segments constituting three visits in the same place.


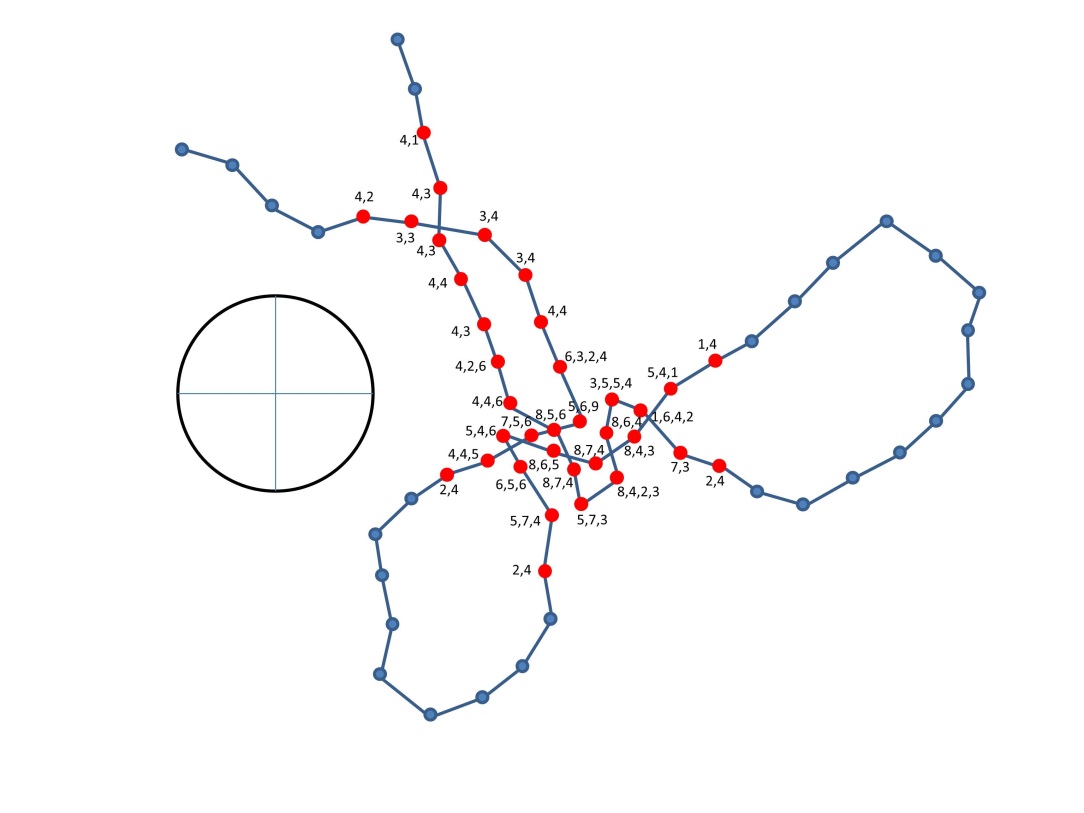

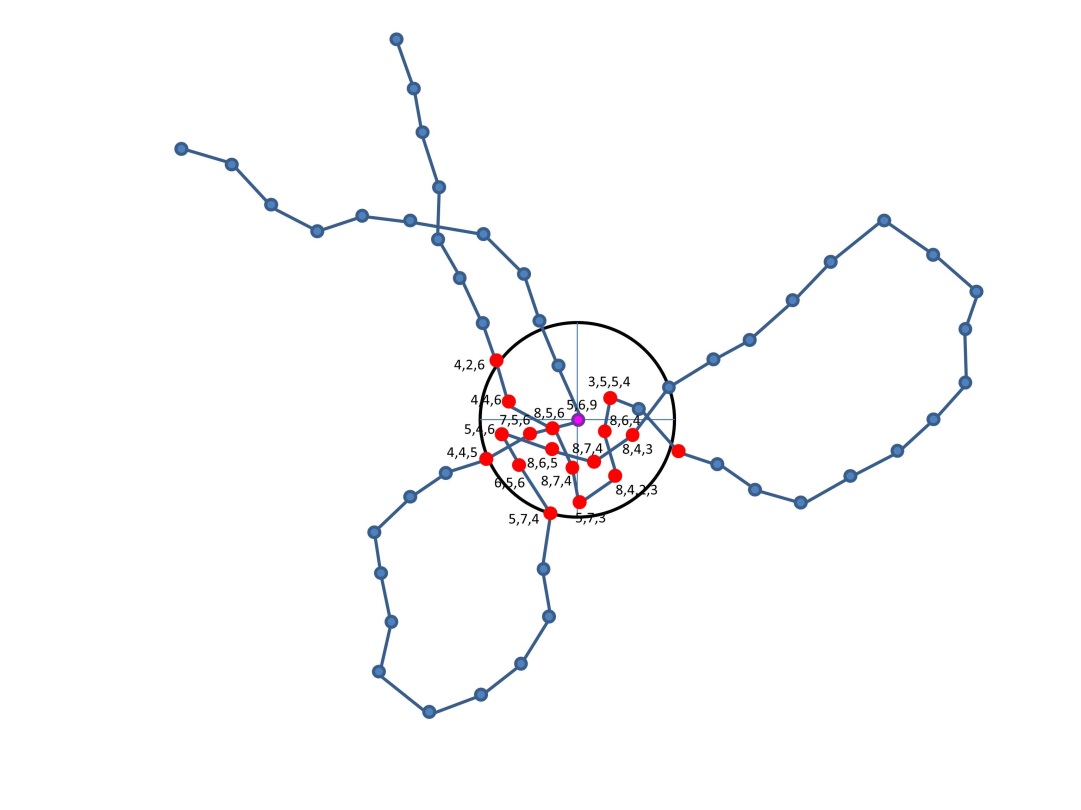

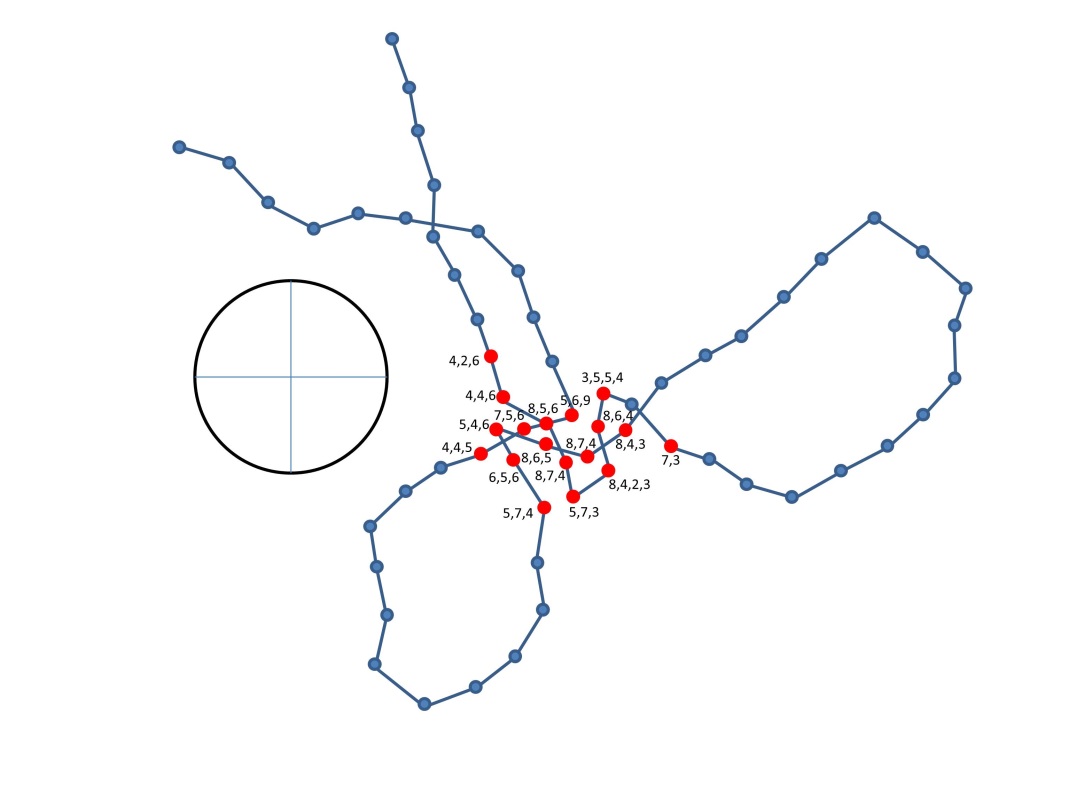


A

B

C

D

E

F


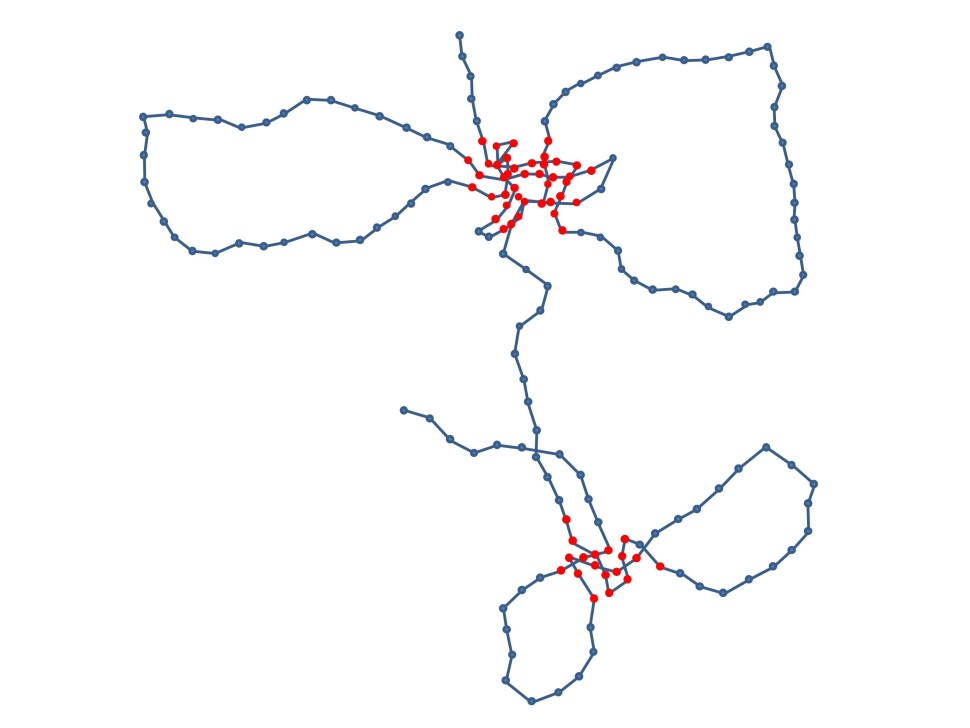

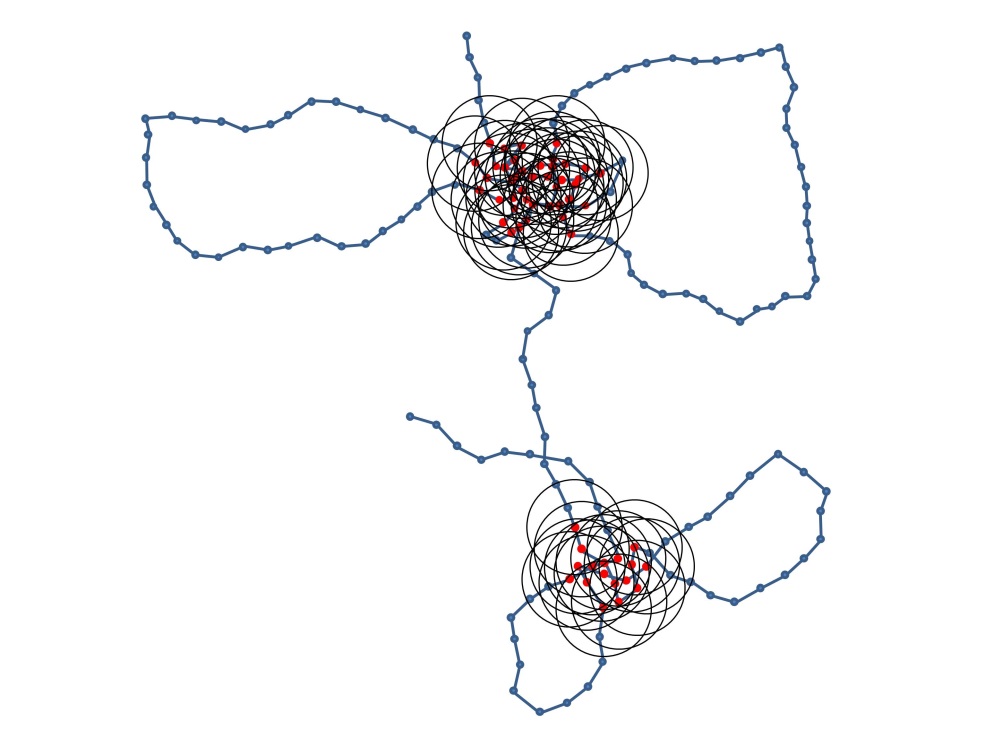

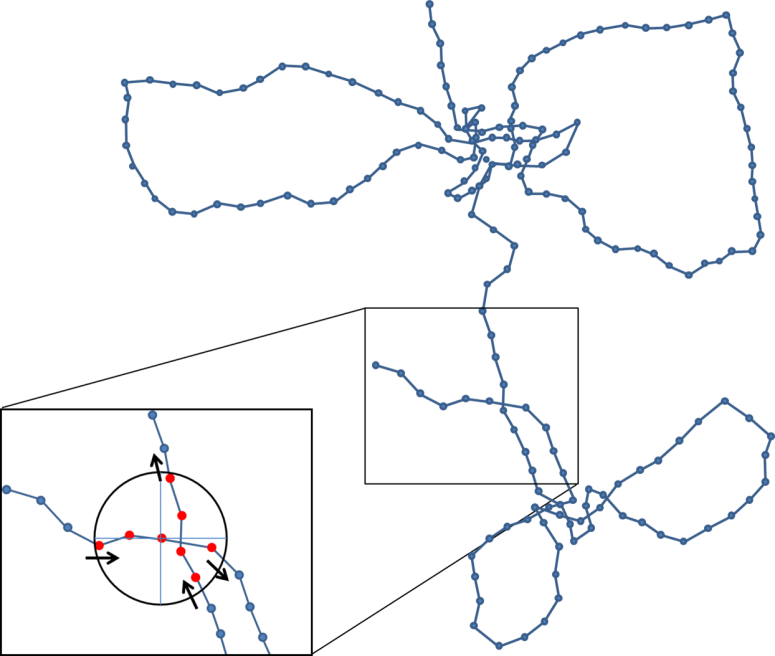


A

C
