# Supplementary material for: Inferring detailed space use from movement paths: A unifying, residence time‐based framework
Source: Ecol Evol. 2017 Sep 12;7(20):8507–14. doi: 10.1002/ece3.3321 (PMC5648670; doi:10.1002/ece3.3321)
Supplement: Supplementary file 2 [file ECE3-7-8507-s002.doc]

**Table S1: Summary results of variance-scale analysis performed on 30 simulation runs for hierarchical ARS on hierarchical patchy resource map and 30 runs for ARS on patchy resource map. The table presents the number of distinct peaks appearing in the curve when the cv(RT) and var(log(RT)) were used, and for each peak its radius. These can be compared with the radius estimated visually (usually a range of radii). Numbers in parentheses are sub-peaks appear near the main peak. * denote a rather weak signature for the peak and ** a poor signature for the peak.**

| Heirarchical ARS on Heirarchical patchy resource map | Run No. | No. of peaks | | Peak 1 | | Peak 2 | | | Peak 3 | | |
| --- | --- | --- | --- | --- | --- | --- | --- | --- | --- | --- | --- |
|  |  | CV | log | CV | log | CV | log | Visually | CV | log | visually |
|  | 1 | 3 | 2 | 10 |  | 75 | 75 | 20-40 | 360 | 380 | 150-200 |
|  | 2 | 3 | 2 |  |  | 30 | 40 | 20-30 | 75 | 100 | 50-80 |
|  | 3 | 3 | 2 | 10 |  | 45 | 55 | 30 | 100 | 170 | 10-150 |
|  | 4 | 4 | 3 | 10 |  | 40 | 50 | 20-50 | 100(200*) | 140(200*) | 150-200 |
|  | 5 | 2 | 1 | 10 |  | 45 | 50 | 15-30 |  |  | 80-120 |
|  | 6 | 3 | 2 | 10 |  | 50 | 60 | 15-40 | 90 | 160 | 50-80 |
|  | 7 | 3 | 2 | 10 |  | 40 | 45 | 15-30 | 140 | 150 | 80-100 |
|  | 8 | 3 | 2 | 10 |  | 40 | 60 | 20-30 | 200 | 250 | 120 |
|  | 9 | 3 | 2 | 10 |  | 45 | 50 | 20-40 | 200** | 200** | 50-120 |
|  | 10 | 3 | 2 | 10 |  | 40 | 45 | 15-30 | 130 | 150 | 80 |
|  | 11 | 3 | 2 | 10 |  | 45 | 45** | 15-30 | 120** | 170 | 80 |
|  | 12 | 3 | 2 | 10 |  | 70 | 70 | 20-40 | 200 | 250 | 100-200 |
|  | 13 | 3 | 2 | 10 |  | 45 | 50 | 30-70 | 120 | 140 | 80-120 |
|  | 14 | 2 | 2 | 10 |  | 40 | 50 | 20-40 |  | 120 | 80-150 |
|  | 15 | 3 | 2 | 10 |  | 50 | 50 | 30 | 140 | 170 | 50-80 |
|  | 16 | 3 | 2 | 10 |  | 35 | 40 | 20-30 | 200* | 200 | 80 |
|  | 17 | 2 | 1 | 10 |  | 60 | 90 | 15-30 |  |  | 80 |
|  | 18 | 3 | 2 | 10 |  | 40 | 40 | 15-40 | 175 | 220 | 100-200 |
|  | 19 | 3 | 2 | 10 |  | 50 | 50 | 20-30 | 120 | 130 | 80-120 |
|  | 20 | 3 | 3 | 10 |  | 60 | 60 | 20-40 | 190 | 120(220) | 100 |
|  | 21 | 2 | 2 |  |  | 45 | 50 | 20-40 | 160 | 180 | 50-100 |
|  | 22 | 3 | 2 | 10 |  | 40 | 45 | 20-40 | 190 | 200 | 80-100 |
|  | 23 | 3 | 2 | 10 |  | 40 | 40 | 15-40 | 160 | 200 | 100-150 |
|  | 24 | 3 | 2 | 10 |  | 40 | 40 | 20-50 | 110 | 150 | 80-100 |
|  | 25 | 3 | 3 | 10 |  | 35 | 40 | 20-40 | 190 | (140*)200 | 100-120 |
|  | 26 | 3 | 2 | 10 |  | 35 | 40 | 20-50 | 110 | 150 | 150 |
|  | 27 | 3 | 2 | 10 |  | 30 | 40 | 20-30 | 150 | 170 | 80-100 |
|  | 28 | 2 | 2 | 10 |  | 50 | 65 | 20-40 |  | 130 | 50-100 |
|  | 29 | 3 | 2 | 10 |  | 40 | 50 | 20-40 | 150 | 170 | 50-100 |
|  | 30 | 3 | 2 | 10 |  | 50 | 60 | 20-40 | 160 | 180 | 80-120 |
| ARS on patchy resource map | 1 | 1 | 1 |  |  | 40 | 40 | 15-30 |  |  |  |
|  | 2 | 3 | 2 | 10 |  | 30 | 40 | 15-40 | 300* | 300* |  |
|  | 3 | 1 | 1 |  |  | 30 | 40 | 15-30 |  |  |  |
|  | 4 | 2 | 2 |  |  | 50 | 60 | 15-30 | 350* | 350** |  |
|  | 5 | 2 | 1 | 10 |  | 30 | 45 | 15-40 |  |  |  |
|  | 6 | 1 | 1 |  |  | 35 | 45 | 15-30 |  |  |  |
|  | 7 | 1 | 1 |  |  | 40 | 50 | 15-40 |  |  |  |
|  | 8 | 1 | 1 |  |  | 35 | 40 | 20-40 |  |  |  |
|  | 9 | 2 | 1 | 10 |  | 40 | 45 | 15-40 |  |  |  |
|  | 10 | 1 | 1 |  |  | 30 | 40 | 15-40 |  |  |  |
|  | 11 | 1 | 1 |  |  | 35 | 40 | 20-30 |  |  |  |
|  | 12 | 2 | 2 |  |  | 35 | 40 | 15-40 | 270* | 280** |  |
|  | 13 | 1 | 1 |  |  | 40 | 45 | 15-40 |  |  |  |
|  | 14 | 1 | 1 |  |  | 40 | 45 | 15-30 |  |  |  |
|  | 15 | 1 | 1 |  |  | 35 | 40 | 15-30 |  |  |  |
|  | 16 | 1 | 1 |  |  | 40 | 40 | 15-30 |  |  |  |
|  | 17 | 1 | 1 |  |  | 30 | 40 | 15-40 |  |  |  |
|  | 18 | 2 | 1 | 10 |  | 40 | 45 | 15-50 |  |  |  |
|  | 19 | 1 | 1 |  |  | 35 | 40 | 15-30 |  |  |  |
|  | 20 | 1 | 1 |  |  | 35 | 45 | 15-30 |  |  |  |
|  | 21 | 1 | 1 |  |  | 40 | 50 | 15-50 |  |  |  |
|  | 22 | 1 | 1 |  |  | 40 | 45 | 15-30 |  |  |  |
|  | 23 | 1 | 1 |  |  | 35 | 45 | 15-30 |  |  |  |
|  | 24 | 1 | 1 |  |  | 30 | 35 | 15-30 |  |  |  |
|  | 25 | 2 | 2 |  |  | 30 | 35 | 15-50 | 360** | 360** |  |
|  | 26 | 2 | 1 | 10 |  | 40 | 45 | 15-40 |  |  |  |
|  | 27 | 2 | 2 |  |  | 35 | 45 | 15-30 | 200-370** | 200-370** |  |
|  | 28 | 1 | 1 |  |  | 40 | 40 | 15-30 |  |  |  |
|  | 29 | 1 | 1 |  |  | 45 | 50 | 20-40 |  |  |  |
|  | 30 | 1 | 1 |  |  | 40 | 40 | 20-40 |  |  |  |

**Table S2: Summary results of revisit analysis performed on 30 simulation runs for ARS on patchy resource map. The table presents the number of distinct ARS places identified when the mean visit duration (MVD) and the total time around the circle were used to filter out locations and choosing representative locations (see main text). These can be compared with the number of ARS places estimated visually. The rates of three errors are presented: distinct places that were erroneously united to one place, and the counting of places not of ARS - stopping places and places with multiple, occasional and short revisits.**

|  | | MVD | | | Total time | | |
| --- | --- | --- | --- | --- | --- | --- | --- |
| Run No. | No. of distinct places identified visually | No. of distinct places identified | United erroneously | Stopping places (not ARS) | No. of distinct places identified | United erroneously | Places with multiple (occasional) short revisits |
| 1 | 14 | 16 | 1 | 3 | 5 | 0 | 0 |
| 2 | 17 | 14 | 2 | 1 | 5 | 3 | 0 |
| 3 | 14 | 14 | 0 | 1 | 9 | 1 | 0 |
| 4 | 14 | 15 | 3 | 4 | 5 | 1 | 0 |
| 5 | 20 | 16 | 0 | 1 | 7 | 2 | 0 |
| 6 | 14 | 13 | 0 | 0 | 10 | 0 | 0 |
| 7 | 17 | 14 | 1 | 0 | 8 | 0 | 0 |
| 8 | 9 | 8 | 0 | 0 | 5 | 0 | 0 |
| 9 | 18 | 16 | 0 | 0 | 7 | 2 | 0 |
| 10 | 13 | 13 | 1 | 2 | 9 | 2 | 1 |
| 11 | 14 | 16 | 0 | 4 | 9 | 0 | 0 |
| 12 | 17 | 12 | 0 | 1 | 4 | 0 | 0 |
| 13 | 18 | 14 | 1 | 0 | 7 | 2 | 0 |
| 14 | 14 | 12 | 0 | 0 | 4 | 1 | 0 |
| 15 | 11 | 13 | 0 | 2 | 6 | 0 | 0 |
| 16 | 14 | 13 | 1 | 0 | 6 | 1 | 0 |
| 17 | 17 | 17 | 0 | 8 |  | 0 | 0 |
| 18 | 12 | 10 | 1 | 1 | 3 | 1 | 0 |
| 19 | 19 | 20 | 0 | 2 | 10 | 1 | 0 |
| 20 | 12 | 11 | 1 | 1 | 5 | 1 | 0 |
| 21 | 18 | 11 | 1 | 0 | 4 | 1 | 0 |
| 22 | 11 | 26 | 1 | 14 | 8 | 1 | 1 |
| 23 | 12 | 11 | 1 | 0 | 4 | 0 | 0 |
| 24 | 16 | 16 | 4 | 3 | 6 | 3 | 0 |
| 25 | 13 | 17 | 1 | 3 | 6 | 0 | 0 |
| 26 | 18 | 14 | 2 | 0 | 8 | 5 | 0 |
| 27 | 16 | 13 | 1 | 0 | 5 | 5 | 0 |
| 28 | 16 | 14 | 2 | 2 | 8 | 4 | 0 |
| 29 | 14 | 11 | 0 | 0 | 6 | 0 | 0 |
| 30 | 15 | 14 | 1 | 1 | 7 | 2 | 0 |
