# Supplementary material for: Inferring detailed space use from movement paths: A unifying, residence time‐based framework
Source: Ecol Evol. 2017 Sep 12;7(20):8507–14. doi: 10.1002/ece3.3321 (PMC5648670; doi:10.1002/ece3.3321)
Supplement: Supplementary file 3 [file ECE3-7-8507-s003.doc]

**Appendix S1: Simulation details**

Simulations were run for 60000 time steps. Resource maps were created on a 1000×1000 cell array. Cells containing a food item had a non-zero value. Three spatial structures of food items were created: scattered, simple patchiness, and two-level hierarchical patchiness (Fig. 1). For the scattered resource map, food items were scattered over the matrix with each cell having a probability of 0.006 to contain a food item. For simple patchiness, patch centers were first scattered with each cell having a probability of 0.000025 to be a patch center. The number of food items in a patch was then drawn from a normal distribution with parameters *μ*=200, *σ*=50. The distance of food items from each patch center was drawn from a normal distribution with parameters *μ*=0, *σ*=15 and their direction was drawn uniformly between 0 and 2π. Patches were thus denser around the center than at the margins. For a hierarchical landscape, clump centers were first scattered with per-cell probability of 0.000008. The number of patches in a clump was 3 or 4, equally probable. The distance of a patch from the clump center was drawn from a normal distribution with parameters *μ*=0, *σ*=15, and its direction was drawn uniformly between 0 and 2π. After being consumed, food items had a renewal time of 600 time steps, thus in the case of patchy resources, patches were depleted at a descending rate while being visited by the forager, and then renewed gradually as food items that had been eaten, reappeared.

In the biased correlated random walk model (see main text), different combinations of *v_b_* and *p* yield the variety of movement behaviors. When *p* is high and *v_b_* is low, the simulated walker performs a directed movement toward the target, or away from it, depending on the sign of *p*. When *v_b_* and *p* are high, the walker performs a concentrated ARS around the target. When *v_b_* is low and *p* is intermediate, the walker performs a widely dispersed exploration behavior: it moves within a wide area around the target and covers this area relatively uniformly (the density of occurrence slightly declines from the location of the target toward the margins, as expected in real home-range behavior).

The three foraging strategies simulated are combinations of these basic movement behaviors. In the first strategy, a simple search, the forager explores the map by moving with direction persistence and overall attraction to the home range center (*v_b_* = 0.1, *p* = 0.5). The forager consumes food items whenever they are within a limited perceptual range of the eight surrounding cells, and in addition performs occasional resting stops for 30-40 time steps. In the second strategy, a simple ARS, the forager explores the map, equipped with a limited perceptual range as described above, until it first encounters a food item. Then, it starts performing an ARS (*v_b_* = 0.8, *p* = 0.8) around the location of this food item. When the next food item is found within a patch, its location becomes the new center of the ARS. If a pre-defined giving-up time of 100 time steps elapses without finding any food item, the forager leaves the patch, and reverts to the exploration mode. The third strategy, the hierarchical ARS, is the same as the simple ARS until the forager leaves a patch. Once leaving a patch, the forager starts searching for another patch in the vicinity of the patch it just left by moving away from it (*v_b_* = 0.1, *p* = -0.7) up to a distance of 80 cell units. Then, if no other patch is found, it starts moving back toward the patch it just left, in a more convoluted path (*v_b_* = 1, *p* = 0.5), until it nears the patch less than 20 cell units. It repeats these forays in different, randomly selected directions, until finding a new patch, or until a pre-defined giving-up time of 300 time steps elapses.
