# Supplementary material for: Inferring detailed space use from movement paths: A unifying, residence time‐based framework
Source: Ecol Evol. 2017 Sep 12;7(20):8507–14. doi: 10.1002/ece3.3321 (PMC5648670; doi:10.1002/ece3.3321)
Supplement: Supplementary file 4 [file ECE3-7-8507-s004.docx]

**Appendix S2:** **Detailed analysis of multiple revisits to the same place**

Locating places the animal visited multiple times and tracing their revisit history can be analyzed further than summarizing the mean visit duration and number of revisits per place. It may be of interest to explore the revisit history over time: periods when a place is visited more often, change in visit durations over time or distribution of between-visit periods. These can all be inferred from the revisit history calculated for each ARS place. We give an example of a simulated trap-lining behavior, where systematic foraging within known food patches is mixed with search forays, and patches are avoided for some period after each visit to accommodate temporary food depletion (food is depleted and replenishes in all of our simulations, see main text and Appendix S1). Figure S1 presents a single realized trajectory from this knowledge-based simulation model, while Figure S2 presents the distribution of between-visit periods for this model versus simple search with ARS. The systematic behavior is expressed by the mode - for the simple search model the frequency of shortest durations between visits is the highest, while for the knowledge-based model, intermediate values are the most frequent. This reflects the intentional avoidance of patches for some period after they were visited. There is also difference in the tail - a shorter tail for the knowledge-based model reflects systematic choosing and visiting within certain patches.


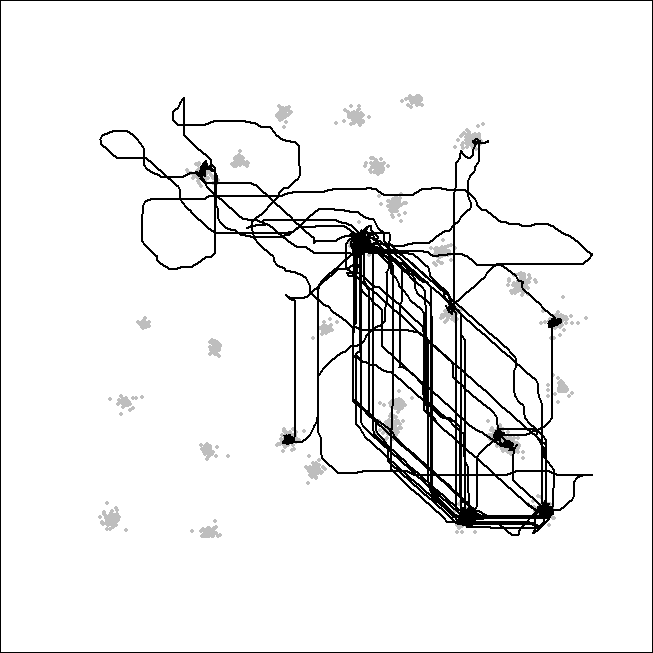


**Figure S1:** a representative trajectory from a simulated trap-lining behavior, where systematic foraging within known food patches is mixed with search forays, and patches are avoided for some period after each visit to accommodate food depletion (food replenishment occurs gradually). All patches and their values are known to the forager, and those having the highest value (number of food items) are given the highest priority.

**Figure S2:** the distribution of between-visit durations for simple search with ARS (A, B) versus systematic trap-lining behavior (C, D). The left column (A,C) shows histograms for the entire data and the right column (B, D) shows histograms for the data after truncating durations longer than 1000 time units. The data accumulated from five simulation runs for each scenario.
